# Supplementary material for: Polygenic scoring accuracy varies across the genetic ancestry continuum
Source: Nature. 2023 May 17;618(7966):774–81. doi: 10.1038/s41586-023-06079-4 (PMC10284707; doi:10.1038/s41586-023-06079-4)
Supplement: Supplementary file 1 — This file contains Supplementary Note, Figs. 1 and 2. Supplementary Note: Theoretical justification for individual PGS accuracy metric. Supplementary Fig. 1: Measured phenotype, PGS estimates, and accuracy varies across the EA GIA in ATLAS. Supplementary Fig. 2: Slope of regressing phenotype on PGS is calibrated across GIA clusters in simulation. See PDF for full legend. [file 41586_2023_6079_MOESM1_ESM.pdf]

nature portfolio

https://doi.org/10.1038/s41586-023-06079-4

Supplementary information

Polygenic scoring accuracy varies across the genetic ancestry continuum

In the format provided by the authors and unedited

Supplementary Information for "Polygenic scoring accuracy varies across the genetic ancestry continuum"

Yi Ding[1], Kangcheng Hou[1], Ziqi Xu[2], Aditya Pimplaskar[1], Ella Petter[2], Kristin Boulier[1], Florian Privé[3], Bjarni J. Vilhjálmsson[3,4,5], Loes M. Olde Loohuis[6,7], Bogdan Pasaniuc[1,7,8,9]

1. Bioinformatics Interdepartmental Program, UCLA, Los Angeles, CA, USA
2. Department of Computer Science, UCLA, Los Angeles, CA, USA
3. National Centre for Register-based Research, Aarhus University, Aarhus, Denmark
4. Bioinformatics Research Centre, Aarhus University, Aarhus, Denmark
5. Novo Nordisk Foundation Center for Genomic Mechanisms of Disease, Broad Institute, Cambridge, MA, USA
6. Center for Neurobehavioral Genetics, Semel Institute for Neuroscience and Human Behavior, David Geffen School of Medicine at UCLA, Los Angeles, CA, USA
7. Department of Human Genetics, David Geffen School of Medicine at UCLA, Los Angeles, CA, USA
8. Department of Computational Medicine, David Geffen School of Medicine at UCLA, Los Angeles, CA, USA
9. Department of Pathology and Laboratory Medicine, David Geffen School of Medicine at UCLA, Los Angeles, CA, USA

Correspondence: Y.D. (yiding920@ucla.edu); B.P. (pasaniuc@ucla.edu).

Table of Contents

Supplementary Note 3

Supplementary Figure 5

Supplementary Table Legends 7

Supplementary Note

In this supplementary note, we demonstrate that the individual PGS accuracy we proposed is as an upper limit for the actual genetic prediction accuracy under two specific conditions (1) when the SNPs included in the model cannot fully explain the total heritability of the trait and (2) when the causal effect sizes are different in training population and testing population. In the following derivation, we need to use equations we developed in the Methods section:

First, the definition of individual PGS accuracy:

$$r_i^2 = \frac{cov_{\beta,D}(g_i, \hat{g}_i)^2}{var_{\beta,D}(g_i) var_{\beta,D}(\hat{g}_i)} = \frac{var_D\left(x_i^T \hat{\beta}\right)^2}{var_\beta\left(x_i^T \beta\right) var_D\left(x_i^T \hat{\beta}\right)} = \frac{var_D\left(x_i^T \hat{\beta}\right)}{var_\beta\left(x_i^T \beta\right)} = 1 - \frac{E_D\left(var_{\beta|D}\left(x_i^T \beta\right)\right)}{var_\beta\left(x_i^T \beta\right)}$$

Second, the property of estimated genetic effects in a random effects model:

$cov_{\beta,D}\left(x_i^T \hat{\beta}, x_i^T \beta\right) = var_D\left(x_i^T \hat{\beta}\right)$ where $\hat{\beta} = E_{\beta|D}(\beta)$

Condition 1: The SNPs included in the model cannot fully explain the total heritability of the trait
We use x_i to represent all causal variants, x_{si} and β_s to denote a subset of SNPs and their effects that are included in the PGS models and x_{-si} and β_{-si} to denote SNPs excluded from the PGS models and their effects, where x_{si} and x_{-si} are independent. In this scenario, the true genetic liability for individual i is $g_i = x_i^T \beta = x_{si}^T \beta_s + x_{-si}^T \beta_{-si}$, the estimated genetic liability is $\hat{g}_i = x_{si}^T \hat{\beta}_s$ and the estimated accuracy is $r_{i,estimate}^2 = \frac{var_D(x_{si}^T \hat{\beta})}{var_\beta(x_{si}^T \beta)}$.

The true genetic prediction accuracy is:

$$\begin{aligned}
r_{i,true}^2 &= \frac{cov_{\beta,D}(g_i, \hat{g}_i)^2}{var_{\beta,D}(g_i) var_{\beta,D}(\hat{g}_i)} \\
&= \frac{cov_{\beta,D}\left(x_{si}^T \beta_s + x_{-si}^T \beta_{-si}, x_{si}^T \hat{\beta}\right)^2}{var_\beta\left(x_{si}^T \beta_s + x_{-si}^T \beta_{-si}\right) var_{\beta,D}\left(x_{si}^T \hat{\beta}\right)} \\
&= \frac{var_D\left(x_{si}^T \hat{\beta}_s\right)^2}{var_\beta\left(x_{si}^T \beta_s + x_{-si}^T \beta_{-s}\right) var_D\left(x_{si}^T \hat{\beta}_s\right)} \\
&= \frac{var_D\left(x_{si}^T \hat{\beta}_s\right)}{var_\beta\left(x_{si}^T \beta_s + x_{-si}^T \beta_{-s}\right)} \\
&< \frac{var_D\left(x_{si}^T \hat{\beta}_s\right)}{var_\beta\left(x_{si}^T \beta_s\right)} = r_{i,estimate}^2
\end{aligned}$$

Intuitively, the discrepancy between true and estimated accuracy comes from the underestimated genetic component in the estimated accuracy.

Condition2: The genetic effects are not consistent between training and testing population

We assume β_1 and β_2 to be two $\mathrm{M} \times 1$ vectors of the true causal effect sizes in the training and testing population, respectively. Each element of the two vectors β_{1m} and β_{2m} are sampled from a distribution

$$\begin{pmatrix} \beta_{1m} \\ \beta_{2m} \end{pmatrix} \sim MVN \begin{pmatrix} \sigma^2 & \rho\sigma^2 \\ \rho\sigma^2 & \sigma^2 \end{pmatrix}$$

Under this condition, the true genetic value for a testing individual is $g_i = x_i^T \beta_2$, the estimated genetic value is $\hat{g}_i = x_i^T \hat{\beta}_1$ and the estimated accuracy is $r_{i,estimate}^2 = \frac{var_D(x_i^T \hat{\beta}_1)}{var_{\beta_1}(x_i^T \beta_1)} = \frac{var_D(x_i^T \hat{\beta}_1)}{x_i^T x_i \sigma^2}$.

The true genetic prediction accuracy can be represented as:

$$r_{i,true}^2 = \frac{cov_{\beta_1,\beta_2,D}(g_i, \hat{g}_i)^2}{var_{\beta_2}(g_i) var_{\beta_1,D}(\hat{g}_i)}$$

$$= \frac{cov_{\beta_1,\beta_2,D}\left(x_i^T \beta_2, x_i^T \hat{\beta}_1\right)^2}{var_{\beta_2}\left(x_i^T \beta_2\right) var_{\beta,D}\left(x_i^T \hat{\beta}_1\right)}$$

$$= \frac{\rho^2 cov_D\left(x_i^T \beta_1, x_i^T \hat{\beta}_1\right)^2}{var_{\beta_2}\left(x_i^T \beta_2\right) var_D\left(x_i^T \hat{\beta}_1\right)}$$

$$= \frac{\rho^2 var_D\left(x_i^T \hat{\beta}_1\right)^2}{var_{\beta_2}\left(x_i^T \beta_2\right) var_D\left(x_i^T \hat{\beta}_1\right)}$$

$$= \frac{\rho^2 var_D(x_i^T \hat{\beta}_1)}{var_{\beta_2}\left(x_i^T \beta_2\right)}$$

$$= \frac{\rho^2 var_D\left(x_i^T \hat{\beta}_1\right)}{x_i^T x_i \sigma^2} = \rho^2 r_{i,estimate}^2$$

As a result, when the genetic correlation between the testing and population is ρ, the ratio of true to estimated genetic prediction accuracy is the squared genetic correlation between training and testing population ρ^2.

Supplementary Figure

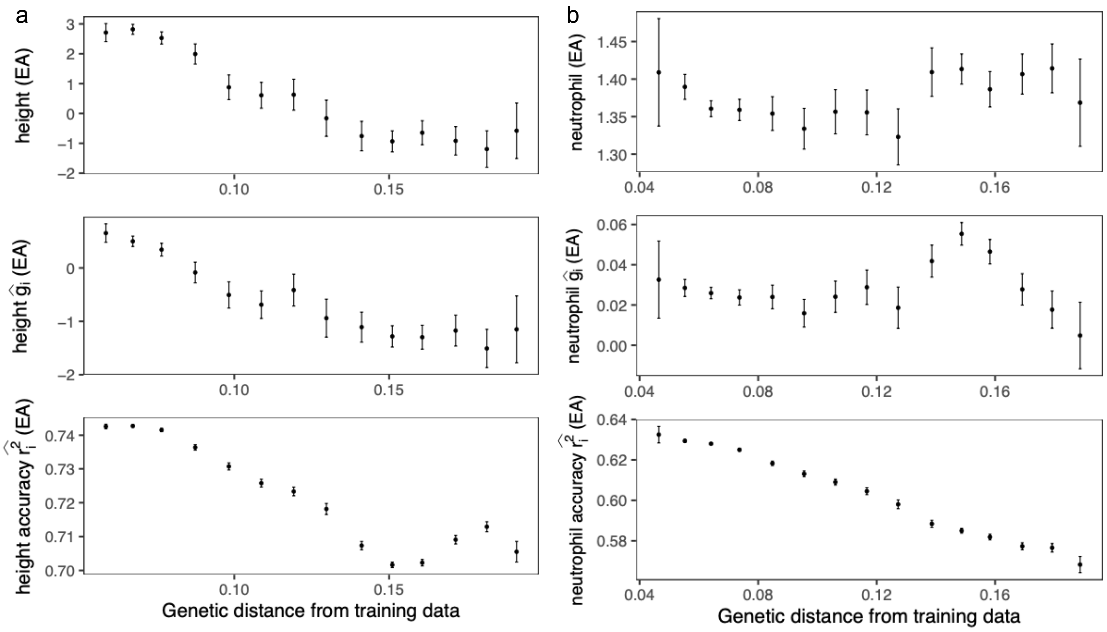

Supplementary Figure 1. Measured phenotype, PGS estimates, and accuracy varies across the EA GIA in ATLAS. a, Variation of height phenotype, PGS estimates and accuracy across different GD bins. **b,** Variation of log neutrophil count phenotype, PGS estimates and accuracy across different GD bins. The 22,380 ATLAS EA GIA individuals are divided into 20 equal-interval GD bins. Bins with fewer than 50 individuals are not shown due to large s.e.m. All panels share the same layout: the x-axis is the average GD within the bin; the y-axis is the average phenotype (top), PGS (middle) and individual PGS accuracy (bottom); the error bars represent +/- 1.96 s.e.m.

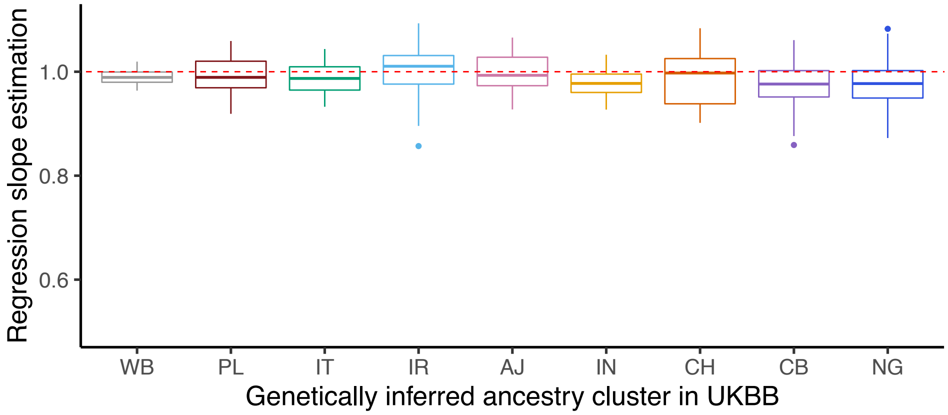

Supplementary Figure 2. Slope of regressing phenotype on PGS is calibrated across GIA clusters in simulation. The PGS model is trained in WB individuals and applied to testing individuals from a diverse genetic background in UKBB. Each boxplot contains 100 points corresponding to the estimated slope by regressing simulated phenotype ($h_g^2 = 0.25, p_{causal} = 0.01$) on PGS estimates for all individuals within the GIA cluster specified by x-axis. The box shows the first, second and third quartile of the 100 slopes, and whiskers extend to the minimum and maximum estimates located within 1.5 × IQR from the first and third quartiles, respectively.

Supplementary Table Legends

Supplementary Table 1. The training sample size, proportion of causal variants and heritability of the 84 traits.

Supplementary Table 2. The correlation between individual PGS accuracy and genetic distance from training data across ATLAS and within each genetic ancestry clusters

Supplementary Table 3. The correlation between individual PGS accuracy and genetic distance from training data across UKBB and within each genetic ancestry clusters

Supplementary Table 4. The correlation between measured phenotype/PGS and genetic distance from training data across UKBB. All p-values were derived from two-sided Pearson correlation tests without adjustment for multiple hypothesis testing.
