# Supplementary material for: Polygenic scoring accuracy varies across the genetic ancestry continuum
Source: Nature. 2023 May 17;618(7966):774–81. doi: 10.1038/s41586-023-06079-4 (PMC10284707; doi:10.1038/s41586-023-06079-4)
Supplement: Supplementary file 2 — Reporting Summary [file 41586_2023_6079_MOESM2_ESM.pdf]

nature portfolio

Corresponding author(s): Yi Ding, Bogdan Pasaniuc

Last updated by author(s): Apr 2, 2023

Reporting Summary

Nature Portfolio wishes to improve the reproducibility of the work that we publish. This form provides structure for consistency and transparency in reporting. For further information on Nature Portfolio policies, see our Editorial Policies and the Editorial Policy Checklist.

Statistics

For all statistical analyses, confirm that the following items are present in the figure legend, table legend, main text, or Methods section.

n/a	Confirmed	
☐	☒	The exact sample size (*n*) for each experimental group/condition, given as a discrete number and unit of measurement
☒	☐	A statement on whether measurements were taken from distinct samples or whether the same sample was measured repeatedly
☐	☒	The statistical test(s) used AND whether they are one- or two-sided<br>*Only common tests should be described solely by name; describe more complex techniques in the Methods section.*
☐	☒	A description of all covariates tested
☐	☒	A description of any assumptions or corrections, such as tests of normality and adjustment for multiple comparisons
☐	☒	A full description of the statistical parameters including central tendency (e.g. means) or other basic estimates (e.g. regression coefficient) AND variation (e.g. standard deviation) or associated estimates of uncertainty (e.g. confidence intervals)
☐	☒	For null hypothesis testing, the test statistic (e.g. *F*, *t*, *r*) with confidence intervals, effect sizes, degrees of freedom and *P* value noted<br>*Give P values as exact values whenever suitable.*
☐	☒	For Bayesian analysis, information on the choice of priors and Markov chain Monte Carlo settings
☒	☐	For hierarchical and complex designs, identification of the appropriate level for tests and full reporting of outcomes
☐	☒	Estimates of effect sizes (e.g. Cohen's *d*, Pearson's *r*), indicating how they were calculated

Our web collection on statistics for biologists contains articles on many of the points above.

Software and code

Policy information about availability of computer code

Data collection	No software is used for data collection.
Data analysis	LDpred2 software implementing individual PRS uncertainty: https://privefl.github.io/bigsnpr/articles/LDpred2.html , https://github.com/yidingdd/individual-pgs-accuracy. Softwares used in this manuscript: bigsnpr(v1.8.1 for LDpred2), flashpca(v2.0), plink(v2.0a3LM), ggplot2(v3.3.5).

For manuscripts utilizing custom algorithms or software that are central to the research but not yet described in published literature, software must be made available to editors and reviewers. We strongly encourage code deposition in a community repository (e.g. GitHub). See the Nature Portfolio guidelines for submitting code & software for further information.

Data

Policy information about availability of data

All manuscripts must include a data availability statement. This statement should provide the following information, where applicable:

- Accession codes, unique identifiers, or web links for publicly available datasets
- A description of any restrictions on data availability
- For clinical datasets or third party data, please ensure that the statement adheres to our policy

The individual-level genotype and phenotype data of UKBB are available by application from http://www.ukbiobank.ac.uk/. Due to privacy concerns, de-identified individual level data for UCLA ATLAS are available to UCLA researchers and can be accessed through the Discovery Data Repository Dashboard (https://

it.uclahealth.org/about/ohia/ohia-products/discovery-data-repository-dashboard-0). Summary ATLAS association statistics are publicly available at https://atlas-phewas.mednet.ucla.edu/. PGS for 84 traits investigated in this manuscript are available at PGS Catalog (PGP000457). MCMC samplings of PGS are available at https://doi.org/10.6084/m9.figshare.22413970.

Human research participants

Policy information about studies involving human research participants and Sex and Gender in Research.

Reporting on sex and gender	Biological sex are used in our study as covariates in training PGS models to avoid potential confounding effects. The finding in our study is applied to both sex.
Population characteristics	The average age of UCLA-ATLAS participants, defined as a participant's age recorded in the EHR as of September 2021, is 55.6 (SD: 17.2) years. Based on biological sex inferred from genotype data, 55% participants are Female and 45% participants are male. We perform PCA to cluster individuals into different genetic ancestry culsters; 61% are inferred to be European Americans, 17% Hispanic/Latino Americans, 2% South Asian Americans, 9% East Asian Americans, 5% African Americans and 6% are unclassified due to lack of reference panel.
Recruitment	Participants are recruited from 18 UCLA Health medical centers, laboratories, and clinics located throughout the greater Los Angeles area. Participants watch a short video outlining the goals of the initiative and document their choice of whether they wish to consent to participation. Our result is not impacted by selection bias if there's any. Full details on recruitment procedure are provided at https://www.uclahealth.org/precision-health/programs/ucla-atlas-community-health-initiative Patient Recruitment and Sample Collection for Precision Health Activities at UCLA is an approved study by the UCLA Institutional Review Board (UCLA IRB). IRB#17-001013
Ethics oversight	UCLA Institutional Review Board

Note that full information on the approval of the study protocol must also be provided in the manuscript.

Field-specific reporting

Please select the one below that is the best fit for your research. If you are not sure, read the appropriate sections before making your selection.

☒ Life sciences ☐ Behavioural & social sciences ☐ Ecological, evolutionary & environmental sciences

For a reference copy of the document with all sections, see nature.com/documents/nr-reporting-summary-flat.pdf

Life sciences study design

All studies must disclose on these points even when the disclosure is negative.

Sample size	We used two dataset in our study: the UK Biobank contains 487409 participants and UCLA-ATLAS contains 36778 participants. We did not collect new dataset for this manuscript. The UK Biobank dataset has a reasonable sample size for training PGS models as it has been widely used for the same purpose by previous publications. The UCLA-ATLAS dataset was solely used for evaluating the PGS performance in our study and its sample size is comparable to previous research studies for the same purpose.
Data exclusions	In UCLA-ATLAS cohort, individuals with >5% missingness in genotype are excluded due to low data quality.
Replication	We successfully replicated our finding that the individual PGS accuracy decays with increased genetic distance from the training data in two datasets: UK Biobank and UCLA-ATLAS.
Randomization	Randomization is not relevant for this study as we didn't assign individuals into different experimental groups.
Blinding	The investigators are blinded to group allocation.

Reporting for specific materials, systems and methods

We require information from authors about some types of materials, experimental systems and methods used in many studies. Here, indicate whether each material, system or method listed is relevant to your study. If you are not sure if a list item applies to your research, read the appropriate section before selecting a response.

Materials & experimental systems

n/a	Involved in the study
☒	☐ Antibodies
☒	☐ Eukaryotic cell lines
☒	☐ Palaeontology and archaeology
☒	☐ Animals and other organisms
☒	☐ Clinical data
☒	☐ Dual use research of concern

Methods

n/a	Involved in the study
☒	☐ ChIP-seq
☒	☐ Flow cytometry
☒	☐ MRI-based neuroimaging
